# Dosimetric Clues to Addressing Urinary Toxicity Following Stereotactic Prostate Radiation Therapy

**DOI:** 10.1016/j.adro.2025.101850

**Published:** 2025-07-03

**Authors:** Michael Cardoso, Matthew Richardson, Phillip Chlap, Sarah Keats, Alan Glyde, Sankar Arumugam, David Pryor, Joseph Bucci, Jarad Martin, Mark Sidhom

**Affiliations:** a Liverpool and Macarthur Cancer Therapy Centres, Sydney, Australia; b South Western Sydney Clinical School, University of New South Wales, Sydney, Australia; c Department of Radiation Oncology, Calvary Mater Newcastle, Waratah, Australia; d Ingham Institute for Applied Medical Research, Sydney, Australia; e Princess Alexandra Hospital, Brisbane, Australia; f St George and Sutherland Clinical School, University of New South Wales, Sydney, Australia; g St George Hospital, Cancer Care Centre, Sydney, Australia; h School of Medicine and Public Health, University of Newcastle, Newcastle, Australia

## Abstract

**Purpose:**

Delayed genitourinary (GU) toxicity is reported following definitive stereotactic body radiation therapy (SBRT) for prostate cancer in 15% to 30% of patients. The purpose of this study is to investigate whether there is a relationship between radiation dose to the bladder and urethra and GU toxicity grade ≥ 2 (National Cancer Institute Common Terminology Criteria for Adverse Events 4.0) in patients treated with SBRT.

**Methods and Materials:**

PROstate Multicenter External beam radioTHErapy Using Stereotactic boost was a phase 2 multicenter trial exploring an SBRT boost of 19 to 20 Gy in 2 fractions to the prostate combined with fractionated external beam radiation therapy as a virtual high-dose-rate brachytherapy boost for patients with prostate cancer. Several bladder and urethral constraints were mandated prospectively. Bladder and the urethral planning organ at risk volume (PRV) dosimetry was correlated with physician-reported GU toxicity for patients ≥ 6 months following SBRT. An association between prior transurethral resection of the prostate (TURP) and urinary toxicity was also examined. Univariant and multivariate analyses were performed.

**Results:**

Of the 151 patients, 87 had complete dosimetric data, and these patients were included in this analysis. In this cohort, 19.5% experienced grade ≥ 2 GU toxicity more than 6 months after stereotactic radiation therapy. On univariate analysis, prostatic urethral length, urethral PRV volume, bladder D2 cc, D5 cc, D10 cc, D15 cc, and bladder V8 were predictive of GU toxicity (all *P* < .05). In the 14 patients who had prior TURP, 6 (43%) experienced GU toxicity compared with 15% for those without prior TURP (*P* = .015). Multivariate analysis showed that prostatic urethral length, urethral PRV volume, bladder 10 cc, and bladder 15 cc remained statistically significant factors predicting GU toxicity.

**Conclusions:**

Prostate SBRT delivered as a virtual high-dose-rate boost is well tolerated overall. However, delayed GU toxicity is experienced by a significant minority of patients. Additional bladder constraints including D10 cc < 17 Gy and D15 cc < 15 Gy may further reduce the risk of delayed GU toxicity. Prior TURP may be a plausible additional risk factor.

## Introduction

Prostate cancer is a common malignancy in men worldwide and image guided radiation therapy is a widely used curative treatment approach.^1^ Conventional external beam radiation therapy (EBRT) without treatment intensification (such as radiation therapy dose escalation and/or androgen deprivation therapy) has resulted in suboptimal cancer outcomes in patients with higher risk prostate cancer.^2^

A high-dose-rate brachytherapy (HDRB) boost is an effective approach to improve prostate cancer relapse-free survival over standard EBRT alone.^3^ A meta-analysis comparing different treatment modalities for prostate cancer found the addition of a brachytherapy boost efficacious for higher risk patients.^4^ Despite this, the utilization of brachytherapy is decreasing for multiple reasons, which includes the expertise required to perform such a technique and the associated cost and time needed. Brachytherapy is an invasive procedure and requires anesthesia and hospital admission. Brachytherapy can also result in a higher risk of urinary toxicity including urinary incontinence and urethral strictures.^5^

Recent technological advances in intrafraction imaging during radiation therapy, and rapid highly conformal dose delivery with volumetric modulated arc therapy, has allowed stereotactic body radiation therapy (SBRT) to evolve into a noninvasive technique to perform dose escalation. Because of these advances, SBRT can now be delivered using linear accelerators which are widely available.

PROstate Multicenter External beam radioTHErapy Using Stereotactic boost (PROMETHEUS) was a phase 2 multicenter clinical trial assessing an SBRT boost to the prostate combined with fractionated EBRT.^6^ Instead of dose escalation using an HDRB boost, the PROMETHEUS trial explored delivering comparable radiation therapy doses with noninvasive SBRT techniques, giving 19 to 20 Gy in 2 fractions delivered 1 week apart, followed by 46 Gy in 23 fractions or 36 Gy in 12 fractions of EBRT^6^ for intermediate and high-risk patients with prostate cancer. With 151 patients accrued, the 5-year disease control was excellent at 94.1%, but the cumulative incidence of grade 2 or higher subacute genitourinary (GU) toxicity of 28.1%.^7^ This married up with results from the PACE-B (PACE - Prostate Advances in Comparative Evidence) trial showing higher grade 2 GU toxicity as well.^8^ A better understanding of how best to mitigate this toxicity would be helpful.

Delayed GU toxicity is reported following definitive SBRT for prostate cancer^9^^,^^10^ and these are usually irritative symptoms which can include urinary frequency, urgency, dysuria, and increased nocturia.^11^ These symptoms typically occur 12 to 18 months after SBRT, and often resolve spontaneously by 24 months.^7^ The purpose of this study is to examine whether in the presence of pre-existing bladder and urethral constraints, there is any relationship between radiation dose to the bladder or urethra and delayed GU radiation therapy-related toxicity grade ≥ 2 [National Cancer Institute Common Terminology Criteria for Adverse Events (CTCAE 4.0)] in patients treated in the PROMETHEUS trial.

## Methods and Materials

### Patient population

Trial details have been previously published.^12^ In summary, men with a diagnosis of intermediate or high-risk prostate adenocarcinoma were eligible, and patients who had locally advanced prostate cancer (such as clinical T4 disease) or nodal disease were excluded. Patients must have been able to have an magnetic resonance imaging (MRI) for radiation therapy planning as well as fiducial seed insertion. Patients who had severe pre-existing urinary symptoms defined as an International Prostate Symptom Score > 20 were excluded. Androgen deprivation therapy was permitted (6 months for unfavorable intermediate risk or 18-24 months for high-risk disease). Before commencement of the PROMETHEUS trial, ethics approval was obtained and written informed consent was obtained from all participants.

### Radiation therapy dose and technique

Before SBRT, patients were planned with a comfortably full bladder and empty rectum. Patients had a rectal displacement device inserted such as the SpaceOAR (Augmenix) or Rectafix (Scanflex Medical AB).

The SBRT clinical target volume (CTV) was the prostate and any extracapsular or seminal vesicle extension seen on MRI planning imaging. The planning target volume was an expansion of 5 mm, except posteriorly where it was 3 mm. Patients were treated with 19 or 20 Gy in 2 fractions delivered 1 week apart, using 1 to 2 coplanar volumetric modulated arc therapy arcs, followed by standard EBRT (46 Gy in 23 fractions or 36 Gy in 12 fractions) commencing 2 weeks later. All patients had MRI fusion for radiation therapy planning, and intrafraction image guidance was mandated.

The entire bladder was contoured from the bladder dome to the bladder neck. In terms of dose constraints for the SBRT component of the treatment for the bladder, the maximum dose to the bladder to 0.1 cc was less than 110% of the target dose (TD). Dose-volume histogram (DVH) parameters, such as bladder V20 (volume receiving 20 Gy), play a valuable role in radiation treatment planning, including radiation therapy toxicity risk estimate. DVH parameters used for the bladder included V20 < 2 cc, V19 < 10 cc, V17 < 15%, and V9 < 50%. As per a previously published protocol by Richardson et al., the urethral position was either delineated using the planning MRI and a 3 mm radial expansion was added to define a urethra planning organ at risk volume (PRV), or the urethra was delineated with the assistance of an indwelling urinary catheter inserted temporarily at the time of simulation.^13^ The maximum dose to 0.1 cc of the urethra PRV was less than 110% of the TD and the V105% TD < 5%.

**Figure 1:**
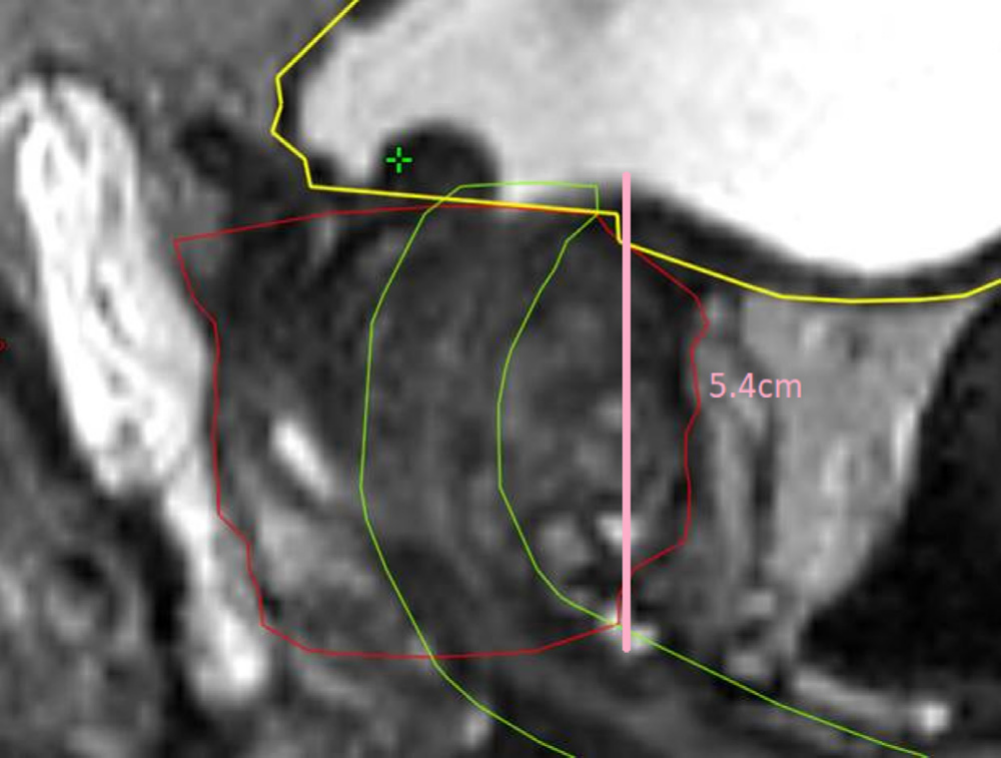
Sagittal image demonstrating the prostatic urethra measurement in pink with the urethra PRV in green, the bladder in yellow and the prostate CTV in red. *Abbreviations:* CTV = clinical target volume; PRV = planning organ at risk volume.

Radiation therapy plans were reviewed to examine organs at risk including the urethra, bladder, and the urethral PRV and this was correlated with physician-reported GU toxicity for patients anytime within 6 months to 60 months following radiation therapy. Metrics used included prostate CTV volume, prostatic urethral length, urethral PRV volume, urethral mean dose, bladder D0.1 cc, D2 cc, D5 cc, D10 cc, D15 cc, V8, V9, V10, V12, V14, V16, V17, and V19. The prostatic urethral length was manually measured from bladder neck to inferior extent of CTV using the measure tool in commercial radiation therapy plan check system MIM (MIM Software Inc., version 6.5.6) in all patients and recorded when reviewing the radiation therapy plans, all other metrics were extracted using PyDicer from the 87 radiation therapy plans based on DVH data.^14^ An example measurement of length of a prostatic urethra is demonstrated in Fig. 1. This measurement of the prostatic urethra was completed by a single investigator.

### Toxicity assessment

Patients were reviewed weekly during radiation therapy treatment. After radiation therapy, urinary toxicity (urinary urgency, dysuria, and frequency) was prospectively graded using the CTCAE v4 at different time points including at the end of EBRT and then at 6 weeks, 6 months, 12 months, 18 months, 24 months, 30 months, 36 months, 48 months, and 60 months post radiation therapy. Toxicity assessment was performed by physicians. An association between prior transurethral resection of the prostate (TURP) and urinary toxicity was also examined. This was done by calculating the percentage of patients who had a prior TURP from within those who had urinary toxicity above grade 2 at any time during follow up after 6 months.

### Statistical analysis

Two groups were created: patients who experienced delayed urinary toxicity of grade 2 and above anytime during the follow-up period, and the patients who did not. The Welch’s *t*-test was used to compare metrics for patients with subacute urinary toxicity of grade 2 and above anytime during the follow up period, and those with only grade 1 or no GU toxicity. The Welch statistic was used to perform a univariant analysis because of unequal sample sizes. We allowed *P* < .05 for the univariate analysis but used *P* < .01 for the multivariate analysis. A general linear model was then used to perform a multivariant analysis using statistically significant metrics (*P* < .01) found during an univariant analysis to conduct a Wilks' Lambda test. All statistical analyses were performed using SPSS software version 22.0 (IBM).

## Results

A total of 87 patients from 5 centers out of 151 patients from the PROMETHEUS trial had radiation therapy plans which could be used for analysis. Of these 87 patients, 17 (19.5%) experienced GU radiation therapy-related toxicity grade ≥ 2, 6 months to 60 months after SBRT. Of these patients who did experience radiation therapy-related GU toxicity, 6 (6.9% of the total) experienced grade 3 toxicity. No patients experienced grade 4 or grade 5 toxicity. One patient was still experiencing grade 2 urinary toxicity at 60 months, but all others had resolved. In the 14 patients who had prior TURP, 6 (43%) experienced GU toxicity compared with 15% for those without prior TURP (*P* = .015). The overall prostate volume in cubic cm was not statistically significantly different between the patients who had urinary toxicity (mean of 48.8 cc) and the patients who did not (mean of 44.7 cc).

On univariant analysis, the prostatic urethral length (cm) and urethral PRV volume (cc), along with mean bladder D2 cc (Gy), D5 cc (Gy), D10 cc (Gy), D15 cc (Gy), and V8 Gy (%) were statistically significant different (*P* < .05) between the patients who experienced GU toxicity and the patients who did not, and these findings are summarized in Table 1 and Table 2. The urethra PRV maximum dose 0.1 cc did not predict GU radiation therapy-related toxicity grade ≥ 2. A multivariant analysis was performed using the Wilks’ Lambda test and it was found the prostatic urethral length, urethral PRV volume, bladder 10 cc and bladder 15 cc remained statistically significant factors predicting for GU toxicity.

**Table 1:** Statistically significant differences between dosimetric factors for patients who had no urinary toxicity and patients with delayed urinary toxicity on univariate analysis

Metric	Patients with no urinary toxicity mean	Patients with urinary toxicity (grade ≥ 2) mean	*P* value
Prostatic urethral length (cm)	4.16 (3.94-4.38)	5.08 (4.56-5.59)	< .01
Urethral PRV volume (cc)	6.0 (5.4-6.7)	8.2 (6.3-10.1)	< .05
Bladder D2 cc (Gy)	20.0 (19.8-20.2)	20.3 (20.1-20.5)	< .05
Bladder D5 cc (Gy)	19.1 (18.7-19.5)	19.8 (19.5-20.1)	< .01
Bladder D10 cc (Gy)	16.9 (16.3-17.6)	18.4 (17.8-19.1)	< .01
Bladder D15 cc (Gy)	14.8 (14.0-15.5)	16.6 (15.6-17.6)	< .01
Bladder V8 Gy (%)	44.0 (38.8-49.2)	55.8 (46.0-65.6)	< .05

*Abbreviations:* PRV = planning organ at risk volume; cc = cubic centimeter.

**Table 2:** Statistically significant differences between dosimetric factors for patients who had no urinary toxicity and patients with urinary toxicity on multivariate analysis

Metric	Patients with no urinary toxicity mean	Patients with urinary toxicity (grade ≥ 2) mean	*P* value
Prostatic urethral length (cm)	4.16 (3.94-4.38)	5.08 (4.56-5.59)	< .01
Urethral PRV volume (cc)	6.0 (5.4-6.7)	8.2 (6.3-10.1)	< .05
Bladder D10 cc (Gy)	16.9 (16.3-17.6)	18.4 (17.8-19.1)	< .01
Bladder D15 cc (Gy)	14.8 (14.0-15.5)	16.6 (15.6-17.6)	< .01

*Abbreviations:* PRV = planning organ at risk volume; cc = cubic centimeter.

**Figure 2:**
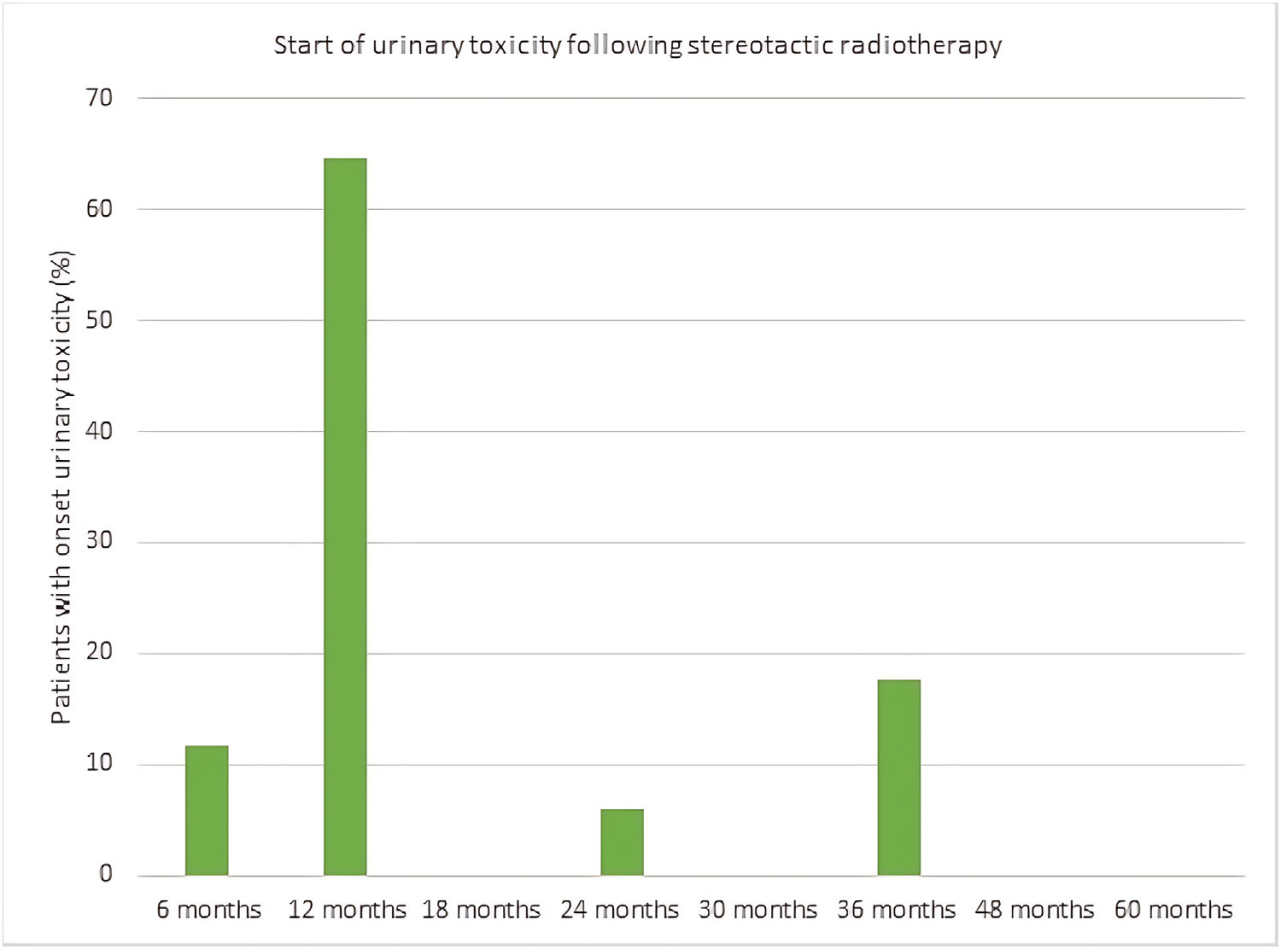
The onset of urinary toxicity at each follow-up visit, expressed as a percentage of all patients who had CTCAE v4 grade ≥ 2 urinary toxicity. *Abbreviations:* CTCAE = National Cancer Institute Common Terminology Criteria for Adverse Events.

The majority of patients who did experience grade ≥ 2 GU toxicity, did so 6-12 months after treatment (76%) and the distribution is shown in Fig. 2.

## Discussion

There are several advantages of using SBRT to deliver dose escalation as an alternative to brachytherapy. These advantages include that SBRT is a noninvasive outpatient procedure which can be delivered with widely available linear accelerators in under 30 minutes without mechanically traumatizing the prostate.

The use of SBRT in prostate cancer treatment is an active area of recent research. Before the PROMETHEUS study, Chen et al. conducted a propensity score-matched analysis comparing a virtual high-dose-rate brachytherapy (VHDRB) cohort (*n* = 131) to a HDRB cohort (*n* = 101). The 10-year biochemical recurrence freedom rate was 85.3% for VHDRB and 74.6% for HDRB (*P* = .3). Grade 3+ GU toxicity rate was 4.6% for VHDRB and 3% for HDRB with no significant difference between the VHDRB and HDRB groups found.^15^ Pasquier et al. used VHDRB of 18 Gy in 3 fractions following a dose of 46 Gy in 23 fractions in 76 patients. This study found the cumulative incidences of GU toxicity of grade ≥ 2 toxicities at 120 months were 1.4% (95% CI, 0.1%-6.6%) with a relapse-free survival of 76.9% (95% CI, 63.1%-86.1%) at 8 years.^16^ Eade et al. conducted a phase 1 sequential dose-escalation study of SBRT which compared 20 Gy, 22 Gy, and 24 Gy to the prostate and 25 Gy, 27.5 Gy, and 30 Gy to the gross tumor volume in 2 fractions, combined with 46 Gy in 23 fractions of external beam radiation in 36 patients. Using this VHDRB strategy, they found the late grade 2 GU toxicity at 2 years was 19.3%.^17^ The PACE-B (PACE - Prostate Advances in Comparative Evidence) study showed at 5 years, the cumulative incidence of late Radiation Therapy Oncology Group grade 2 or higher GU toxicity was 26.9% (95% CI, 22.8-31.5) with SBRT, highlighting that delayed toxicity is not just seen in VHDRB but also in SBRT monotherapy.^8^

Moving forward, the VHDR regimen is being explored in several randomized trials including the Trans Tasman Radiation Oncology Group (TROG) 18-01 NINJA trial (Novel Integration of New prostate radiation schedules with adJuvant Androgen deprivation for patients with intermediate or low-high risk prostate cancer), which is a phase 3 randomized clinical trial assessing the use of a stereotactic boost using 20 Gy in 2 fractions with EBRT versus a 5 fraction SBRT monotherapy approach.^18^ It can be seen from these studies that VHDRB is an effective treatment for prostate cancer but one consistent issue arising from these studies is GU toxicity.

Through analysis of the SBRT treatments delivered on the PROMETHEUS clinical trial, we have demonstrated additional dosimetric factors beyond those already mandated within the protocol impacting urinary toxicity. In this cohort of patients in the PROMETHEUS trial, we found that most patients who experienced GU toxicity after 6 months had it begin to occur by 12 months (76%). This was usually characterized by urinary urgency, dysuria, and frequency, often resolving by the 24 months mark.^7^

It is thought that GU toxicity is multifactorial and can be contingent on other influences other than bladder and urethral radiation therapy dose alone.^19^ Despite this, radiation therapy dose to the bladder and urethra does matter meaningfully and by reducing the radiation therapy dose to the bladder and the urethra, it can be assumed that the chances of developing grade 2 or greater urinary-related toxicity would be reduced. Developing evidence implies that the prostatic urethra is a radiation-sensitive structure with early data suggesting by limiting dose to the urethra, the amount of GU toxicity can be limited.^11^ From our results, possible bladder constraints to use in future studies would include D10 cc < 17 Gy and D15 cc < 15 Gy. Although clearly the lower the doses that could be achieved, the lower the expected risk.

Other studies have also shown that the radiation therapy dose to subregions of the bladder and urethra can be predictive of urinary toxicity following prostate radiation therapy.^20^ We did not find that a larger prostate size predicted for urinary toxicity with statistical significance. A previous SBRT study has shown that larger prostates (> 50 cm^3^) had worse GU toxicity outcomes compared to smaller prostates.^21^ In our study, patients who had prostates larger than 50 cc appeared to have increased rates of GU toxicity, but this did not reach statistical significance independent of urethral and bladder dosimetry. Regardless, the overall differences in prostate volume were small and would not be a reason to preclude patients from SBRT.

Fourteen patients had prior TURP and 6 (43%) experienced GU radiation therapy-related toxicity which is statistically significantly higher than the average rate of GU toxicity of 15% in the non-TURP cohort. This is indicative of prior TURP being a risk factor for developing GU toxicity following stereotactic radiation therapy. When using stereotactic radiation therapy treatment for prostate cancer, the data regarding the impact of TURP on GU toxicity are limited. A previous phase 1 dose-escalation study of stereotactic radiation therapy for low and intermediate risk prostate cancer also showed that prior TURP was found to be associated with more GU toxicity.^22^ In contrast to this, Pepin et al. found that GU toxicity rates in patients who had prior TURP treated with stereotactic radiation therapy had rates that were comparable to patients who were treated with conventionally fractionated radiation therapy.^23^ Given the underlying uropathology and increased surface area of the TURP cavity, it is plausible that TURP would be a risk factor for increased toxicity risk post SBRT. We would recommend that patients with prior TURP should be counseled regarding the possible increased probability of experiencing GU radiation therapy-related toxicity with stereotactic radiation therapy.^7^

This study did have several strengths. One is the large sample size that could be analyzed with repeated measurements of GU radiation therapy-related toxicity measured over time which increased the statistical power of our findings. Because of the relatively large cohort of patients, we were able to observe that patients who experienced urinary toxicity had higher doses to the bladder and urethra. The radiation therapy plans were stored centrally and so additional dosimetric factors could be analyzed.

A limitation of this study is that the delineation of the urethra was often difficult in the absence of a urinary catheter, even with the use of a planning MRI scan. This could result in interobserver variability and making manual measurements of the urethra less accurate for analysis. Also, a small number of these patients in this study had prior TURP and the TURP cavity is not directly equivalent to the structure of the prostatic urethra, potentially introducing error in urethral measurements. It is also possible that because of the tight dose constraints set in the PROMETHEUS trial, the range of treatment plan variation was reduced and therefore the ability to detect some relationships between dosimetric factors and the occurrence of GU radiation therapy-related toxicity post treatment was also reduced. A further limitation was that detailed data on patient factors which could potentially contribute to GU toxicity such as comorbidities were not available. An additional limitation is interobserver variability in capturing CTCAE toxicity grades. With 5 centers included in the trial over a 5-year time period, there were multiple providers filling out the CTCAE toxicity rates, and this could potentially contribute to unanticipated observer or institutional biases.

## Conclusion

Prostate stereotactic radiation therapy appears to be well tolerated overall, but GU toxicity has been observed in this cohort of patients. A transient peak in GU toxicity began at 6 months which subsequently resolved in most patients. A strict dose constraint on the bladder D2 cc, D5 cc, D10 cc, bladder D15 cc, and bladder V8 should be used in future studies to prevent GU toxicity. In addition to maximal urethral dose constraints used in our protocol, we suggest additional bladder constraints including D10 cc < 17 Gy and D15 cc < 15 Gy. We observed increased GU toxicity rates in patients with prior TURP and caution should be used with these patients.

## Disclosures

The authors declare that they have no known competing financial interests or personal relationships that could have appeared to influence the work reported in this paper.
